# Connective tissue growth factor-specific monoclonal antibody inhibits growth of malignant mesothelioma in an orthotopic mouse model

**DOI:** 10.18632/oncotarget.24892

**Published:** 2018-04-06

**Authors:** Yuuki Ohara, Shan Hwu Chew, Nobuaki Misawa, Shenqi Wang, Daiki Somiya, Kae Nakamura, Hiroaki Kajiyama, Fumitaka Kikkawa, Yuta Tsuyuki, Li Jiang, Kyoko Yamashita, Yoshitaka Sekido, Kenneth E. Lipson, Shinya Toyokuni

**Affiliations:** ^1^ Department of Pathology and Biological Responses, Nagoya University Graduate School of Medicine, Nagoya 466-8550, Japan; ^2^ Department of Obstetrics and Gynecology, Nagoya University Graduate School of Medicine, Nagoya 466-8550, Japan; ^3^ Department of Pathology and Laboratory Medicine, Nagoya University Graduate School of Medicine, Nagoya 466-8550, Japan; ^4^ Division of Molecular Oncology, Aichi Cancer Center Research Institute, Nagoya 464-8681, Japan; ^5^ FibroGen, Inc., San Francisco, CA 94158, USA; ^6^ Sydney Medical School, The University of Sydney, Sydney 2006, Australia

**Keywords:** connective tissue growth factor (CTGF), malignant mesothelioma, molecular target therapy, FG-3019 (pamrevlumab), tumor microenvironment

## Abstract

Malignant mesothelioma is an aggressive neoplasm with no particularly effective treatments. We previously reported that overexpression of connective tissue growth factor (CTGF/CCN2) promotes mesothelioma growth, thus suggesting it as a novel molecular target. A human monoclonal antibody that antagonizes CTGF (FG-3019, pamrevlumab) attenuates malignant properties of different kinds of human cancers and is currently under clinical trial for the treatment of pancreatic cancer. This study reports the effects of FG-3019 on human mesothelioma *in vitro* and *in vivo*. We analyzed the effects of FG-3019 on the proliferation, apoptosis, migration/invasion, adhesion and anchorage-independent growth in three human mesothelioma cell lines, among which ACC-MESO-4 was most efficiently blocked with FG-3019 and was chosen for *in vivo* experiments. We also evaluated the coexistent effects of fibroblasts on mesothelioma *in vitro*, which are also known to produce CTGF in various pathologic situations. Coexistent fibroblasts in transwell systems remarkably promoted the proliferation and migration/invasion of mesothelioma cells. In orthotopic nude mice model, FG-3019 significantly inhibited mesothelioma growth. Histological analyses revealed that FG-3019 not only inhibited the proliferation but also induced apoptosis in both mesothelioma cells and fibroblasts. Our data suggest that FG-3019 antibody therapy could be a novel additional choice for the treatment of mesothelioma.

## INTRODUCTION

Malignant mesothelioma is a tumor caused primarily by asbestos exposure [1, 2]. Asbestos is now recognized as a carcinogen and its use has been legally prohibited in most developed countries. However, because it takes 30-40 years to develop mesothelioma after asbestos inhalation, the incidence of mesothelioma is expected to rise in the coming decades [3, 4]. In Europe, its peak incidence is predicted to be in 2015-2020 whereas there is a delay in the peak in Japan and in other non-Western countries [3]. Old buildings built before the regulation harbor asbestos [2] and may cause scattering of asbestos by earthquake [5], tsunami, storm or man-made disaster [6]. Mesothelioma is one of the most aggressive tumors and its median survival is expected as 4-18 months for pleural forms [1]. There are three distinct histologic subtypes: epithelioid, biphasic and sarcomatoid [1]. Clinically, patients with the sarcomatoid subtype present the poorest prognosis with remarkably short survival [7]. It is thus necessary to understand the molecular mechanisms that regulate mesothelial carcinogenesis [8, 9] and mesothelioma progression [10], and to develop molecularly targeted drugs to improve the patients’ prognosis.

We have previously reported that the levels of connective tissue growth factor (CTGF/CCN2) correlate with the malignant behavior of mesothelioma cells [10, 11]. CTGF, a member of the CCN cysteine-rich family, is a 36-38 kDa multifunctional secretory protein, consisting of four modular domains. Modules 1, 2 and 3 have homology to insulin-like growth factor-binding proteins, *von Willebrand* factor type C repeat and thrombospondin type 1 repeat, respectively. Module 4 contains a cysteine knot motif [12–15]. Each module of CTGF is thought to regulate cellular responses by activating/inhibiting cytokines, growth factors (e.g., transforming growth factor-β, TGF-β; bone morphogenetic protein, BMP; vascular endothelial growth factor, VEGF) [16], proteins in the extracellular matrix and cellular receptors such as low-density lipoprotein receptor-related proteins (LRP1 and LRP6, a co-receptor of Wnt) and integrins [12–15]. Among the major cellular responses, CTGF activates angiogenesis [17–19], cellular proliferation, fibrosis, inflammation, epithelial-to-mesenchymal transition and tumor invasion/metastasis [10–13] whereas it usually inhibits apoptosis [12–15]. Conversely, *CTGF* expression is regulated by many different factors and physiological conditions, including TGF-β, hypoxia, VEGF, BMP and Wnt [12–15, 20]. Based on these interactions with various factors, CTGF has been involved in multiple pathogeneses in an autocrine or paracrine manner [12, 13, 21]. *CTGF* overexpression is reported in several distinct human diseases, including idiopathic pulmonary fibrosis (IPF), liver fibrosis/cirrhosis, nephropathy/glomerulosclerosis, pancreatic ductal adenocarcinoma (PDAC), malignant melanoma and ovarian cancer [12–15] in association with progression of the disease and/or poor survival [10–14]. Of note, elevated *CTGF* expression has been reported not only in tumor cells, but also in stromal cells [12–15]. RNA*i*-mediated silencing of *CTGF* expression or monoclonal antibody against CTGF has been reported to attenuate malignant properties of several different tumors [22–26]. FG-3019 (pamrevlumab) is a human antibody specific for CTGF, and is currently under clinical trials for the treatment of IPF [27] and PDAC [28], which revealed improved pulmonary fibrosis in IPF and prolonged survival in PDAC. In the present study, we, for the first time, evaluated the effects of FG-3019 on human mesothelioma cells *in vitro* and *in vivo*.

Cisplatin (CDDP), pemetrexed (PEM), raltitrexed, gemcitabine and etoposide have been used for the treatment of mesothelioma [29]. The current first-line chemotherapy is the combination of CDDP and PEM, with an improvement in median survival of about 3 months (CDDP alone 9.3 months *versus* CDDP + PEM 12.1 months) [30]. Mesothelioma is often diagnosed at an advanced stage in aged population, who therefore may not tolerate the regimen of CDDP + PEM. In frail, elderly patients, a single agent regimen (PEM) has been used, not only in an advanced-stage non-small cell lung cancer [31], but also in mesothelioma [32]. We thus selected single PEM chemotherapy to evaluate its synergistic effect by the use of FG-3019 and also evaluated the role of fibroblasts herein. In the present study, FG-3019 was scarcely effective in conventional 2-dimensional cell culture but was significantly effective in an orthotopic nude mice model.

## RESULTS

### Variations in CTGF levels in human mesothelioma cell lines

**Figure 1:**
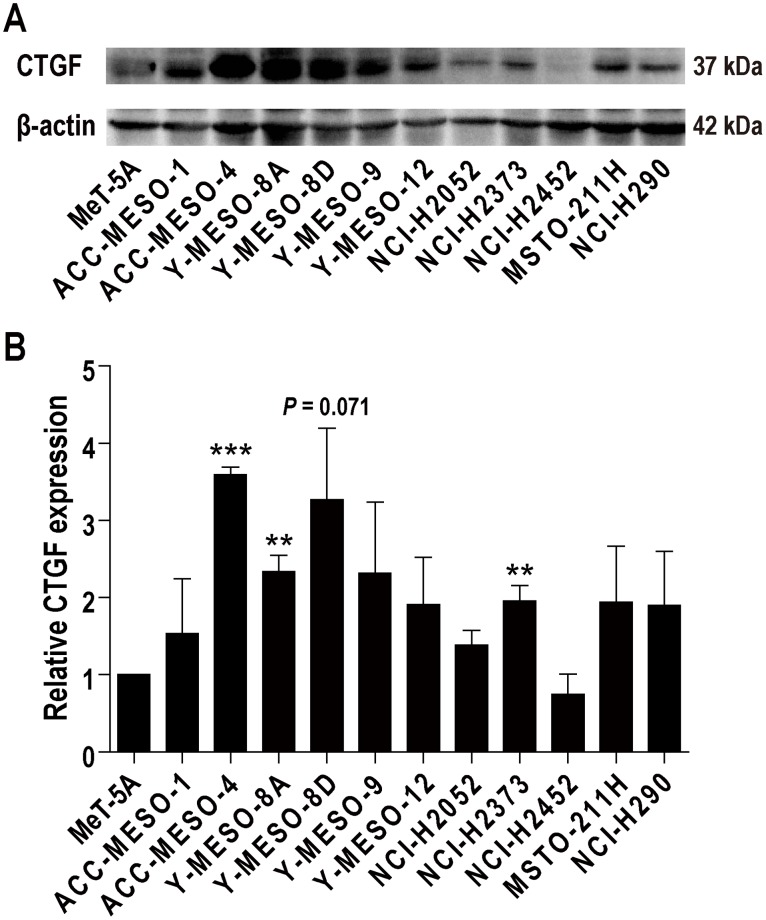
CTGF expression in human mesothelioma cell lines **(A)** Western blot analysis. Antibody 14939 (Santa Cruz Biotechnology; 1:200) was used to detect CTGF at 36-38 kDa. All the cell lines examined expressed CTGF, but several cell lines expressed low levels of CTGF, irrespective of histological subtypes. Three cell lines (ACC-MESO-4, Y-MESO-8D and NCI-H290) were chosen for the following experiments. ACC-MESO-4 and NCI-H290 are epithelioid subtype, and Y-MESO-8D is sarcomatoid subtype. **(B)** Semiquantitative analysis of western blot analysis. Relative CTGF expression in comparison to MeT-5A was calculated with ImageJ. N = 3; means ± SEM, ^**^P < 0.01, ^***^P < 0.001.

Previous studies revealed that normal mesothelial cells *in situ* express little CTGF but mesothelioma cells express high levels of CTGF, which is associated with the malignant characteristics [10, 11]. We first performed western blot analysis to confirm which human mesothelioma cell lines express high levels of CTGF. All the cell lines examined expressed CTGF, but several cell lines expressed low levels of CTGF, irrespective of histological subtypes (Figure 1A and 1B). Based on previous pancreatic cancer studies using FG-3019 [22, 23, 25], we chose the cell lines which expressed higher CTGF levels; ACC-MESO-4 (epithelioid type) with high expression, and Y-MESO-8D (sarcomatoid type) and NCI-H290 (epithelioid type), with moderate to low expression.

### Evaluation of *in vitro* effects of PEM or FG-3019 monotherapy and combination treatment on mesothelioma cell lines

**Figure 2:**
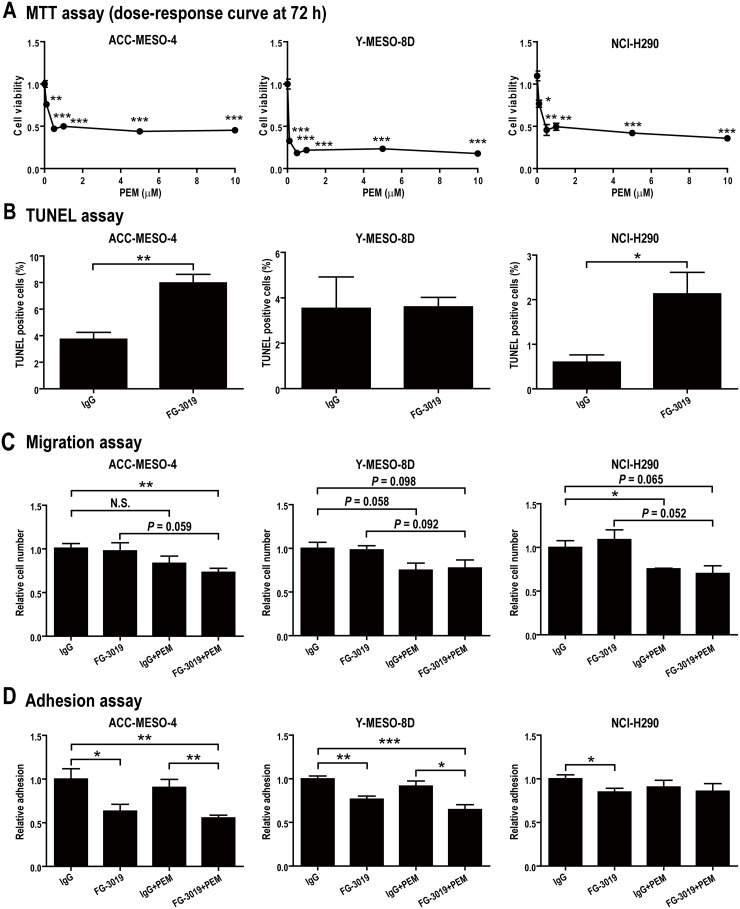
*In vitro* effects of FG-3019 on mesothelioma cell lines in combination with PEM **(A)** Effect of PEM on cell viability. Mesothelioma cells were incubated for 72 h in the medium containing PEM (0-10 μM). **(B)** Apoptosis assay. Apoptotic cells were measured by TUNEL assay after 48 h-incubation in the presence of agents. FG-3019 alone induced apoptosis in ACC-MESO-4 and NCI-H290. **(C)** Migration assay. Migration assay using cell culture insert was performed after 24 h-incubation with agents. As compared with IgG group, migration was inhibited only in FG-3019 + PEM group, indicating that FG-3019 enhanced the effect of PEM. **(D)** Adhesion assay. Attached cells on Matrigel matrix-coated plate were measured with MTT assay. FG-3019 alone inhibited cell adhesion in the three cell lines. N = 4 in (C), N = 5 in (D), N = 3 in the others; means ± SEM, ^*^P < 0.05, ^**^P < 0.01, ^***^P < 0.005. (B)-(D), IgG/FG-3019: 100 μg/ml, PEM: 1 μM. PEM, pemetrexed; N.S., not significant.

We evaluated the ability of PEM to inhibit viability of the mesothelioma cell lines, using the MTT assay (Figure 2A). The cytotoxic effect of PEM reached a maximum at 0.5-1 μM in each line. After administration of a standard PEM dose (500 mg/m^2^) to humans, the maximal plasma concentration was reported to be > 200 μM, which rapidly decreased at 8 h to 8 μM and at 24 h to 0.2 μM [33]. In some previous *in vitro* studies, high concentrations of PEM (> 20 μM) were used for mesothelioma [34, 35] whereas low concentrations of PEM (< 0.5 μM) were used in others for mesothelioma and non-small cell lung cancer [36, 37]. We hypothesized that extreme concentrations are not optimal for experiments to assess the synergistic effect with FG-3019. Therefore, we used 1 μM, which is the minimal concentration that consistently confers maximal toxicity to mesothelioma cells. To determine if FG-3019 (100 μg/ml) inhibits the tumorigenic properties of mesothelioma cells alone, or if it enhances the activity of PEM (1 μM), the response of mesothelioma cells to the therapeutic agents was monitored in several different assays. The concentration of FG-3019 was selected based on a previous report [22]. Non-specific human IgG (100 μg/ml) was used as a control for FG-3019.

We evaluated the proliferation, apoptosis, migration, invasion and anchorage-independent cell growth of the three mesothelioma cell lines under the agent concentrations determined above (IgG/FG-3019: 100 μg/ml, PEM: 1 μM). For proliferation, FG-3019 exhibited no apparent blocking effect in comparison to the IgG control group. Similarly, addition of FG-3019 to PEM treatment showed no enhanced activity on any of the cell lines examined (Supplementary Figure 1A). In the Terminal deoxynucleotidyl transferase dUTP nick end labeling (TUNEL) assay to detect apoptosis, FG-3019 alone induced apoptosis in ACC-MESO-4 (*P* < 0.01) and NCI-H290 (*P* < 0.05) but not in Y-MESO-8D (Figure 2B). There was no significant difference between IgG + PEM and FG-3019 + PEM (Supplementary Figure 1B) although PEM induced a higher percentage of apoptosis than FG-3019 monotherapy (Figure 2B). In the migration assay, only the FG-3019 + PEM group using ACC-MESO-4 cells exhibited decreased cell migration, indicating an increase in response by this combination (*P* < 0.01; Figure 2C). There was no difference in the results of invasion assay (Supplementary Figure 1C) whether cells were treated with FG-3019 alone or in combination with PEM. In the adhesion assay, FG-3019 alone inhibited cell adhesion, which was significantly enhanced in combination with PEM (*P* < 0.05; Figure 2D). In the soft agar colony formation assay, FG-3019 alone marginally inhibited the anchorage-independent growth of NCI-H290 but not that of Y-MESO-8D (Supplementary Figure 1D). No colonies were formed in any of the cell lines when PEM was added. ACC-MESO-4 cells formed no colonies even without any treatment. ACC-MESO-4 cells were more potently inhibited by PEM in the invasion assay (Supplementary Figure 1C) than in the migration assay (Figure 2C) in comparison to the other cell lines. These results suggest that ACC-MESO-4 is inferior to the other cell lines in adaptation to an anchorage-independent environment and thus may be more susceptible to FG-3019. Accordingly, we chose ACC-MESO-4 for use in the xenograft model.

### Fibroblasts significantly promote proliferation and migration/invasion of mesothelioma cells *in vitro*

**Figure 3:**
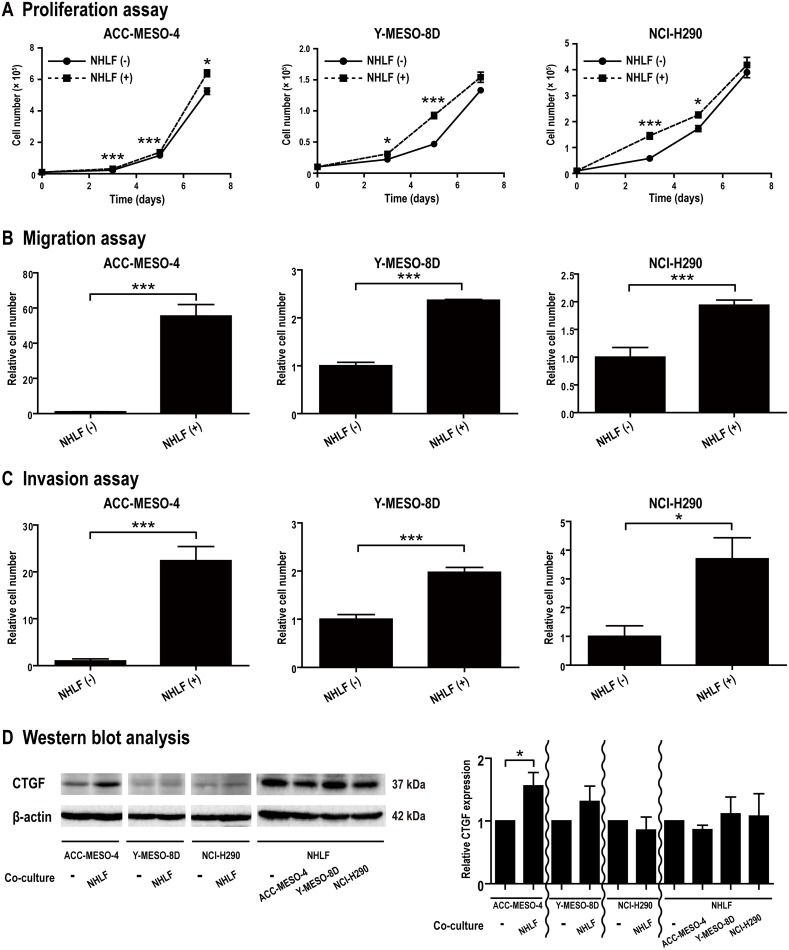
Co-culture experiments using mesothelioma cell lines and fibroblasts **(A)** Proliferation assay. NHLF promoted proliferation of mesothelioma cells. **(B, C)** Migration/Invasion assay. NHLF promoted migration and invasion of mesothelioma cells. **(D)** Western blot analysis. CTGF expression in ACC-MESO-4 was significantly increased upon co-culture. NHLF also expressed CTGF. N = 3 for each group in (A)-(C) and N = 4 in (D); means ± SEM, ^*^P < 0.05, ^**^P < 0.01, ^***^P < 0.005. NHLF, normal human lung fibroblast.

Although stromal cells express CTGF in diseases, such as IPF and liver cirrhosis [12–15], their role in mesothelioma has not yet been elucidated. Therefore, we performed co-culture experiments using normal human lung fibroblast (NHLF) and three mesothelioma cell lines to evaluate the effects of fibroblasts on mesothelioma cells. Proliferation, migration and invasion of mesothelioma cells were significantly promoted by the presence of NHLF in all the three cell lines (*P* < 0.05; Figure 3A-3C). The co-culture effect in transwell permeable support systems for migration and invasion was greater in ACC-MESO-4 than in the other cell lines; ACC-MESO-4, >20-fold; Y-MESO-8D and NCI-H290, ~2-fold (Figure 3B and 3C). In western blot analysis, CTGF expression in ACC-MESO-4 was significantly increased upon co-culture and NHLF also expressed CTGF (*P* < 0.05; Figure 3D). Migration of ACC-MESO-4 cells co-cultured with NHLF could not be inhibited by monotherapy with either FG-3019 or PEM, or by their combination. Migration of both of the other cell lines could be inhibited by PEM when they were co-cultured with NHLF, while FG-3019 was only effective at inhibiting the migration of NCI-H290 co-cultured with NHLF (*P* < 0.05; Supplementary Figure 2A). Co-culture did not alter the lack of response to FG-3019 monotherapy for invasion of any of the cell lines (Supplementary Figures 1C and 2B). Co-culture of ACC-MESO-4 cells with NHLF decreased the sensitivity to PEM for inhibition of invasion (Supplementary Figures 1C and 2B). Proliferation of NHLF was extremely slow with a doubling time of ~400 h. Therefore, the *in vitro* experiments were short enough to ignore the growth of NHLF.

In order to confirm direct effects of CTGF on mesothelioma cells, experiments using recombinant human CTGF (rhCTGF) were performed (Supplementary Figure 3). Previous studies reported that rhCTGF promotes proliferation/invasion of various cells, including pancreatic cancer cells [23] and osteoblasts [38]. We used comparable concentrations of rhCTGF (100 - 200 ng/ml). rhCTGF promoted proliferation of ACC-MESO-4 and NCI-H290 (*P* < 0.05; Supplementary Figure 3A). The migration or invasion of each of the cell lines was enhanced by rhCTGF, although statistical significance was not achieved for NCI-H290 cells in the migration assay and for Y-MESO-8D in the invasion assay (Supplementary Figure 3B and 3C). These effects are comparable to those in the prior reports [23, 38]. Therefore, these data suggest that secretion of CTGF contributes to the effects of NHLF co-culture.

### FG-3019 attenuates mesothelioma growth *in vivo* by inhibiting proliferation and inducing apoptosis in an orthotopic xenograft model with enhanced effect in combination with PEM

**Figure 4:**
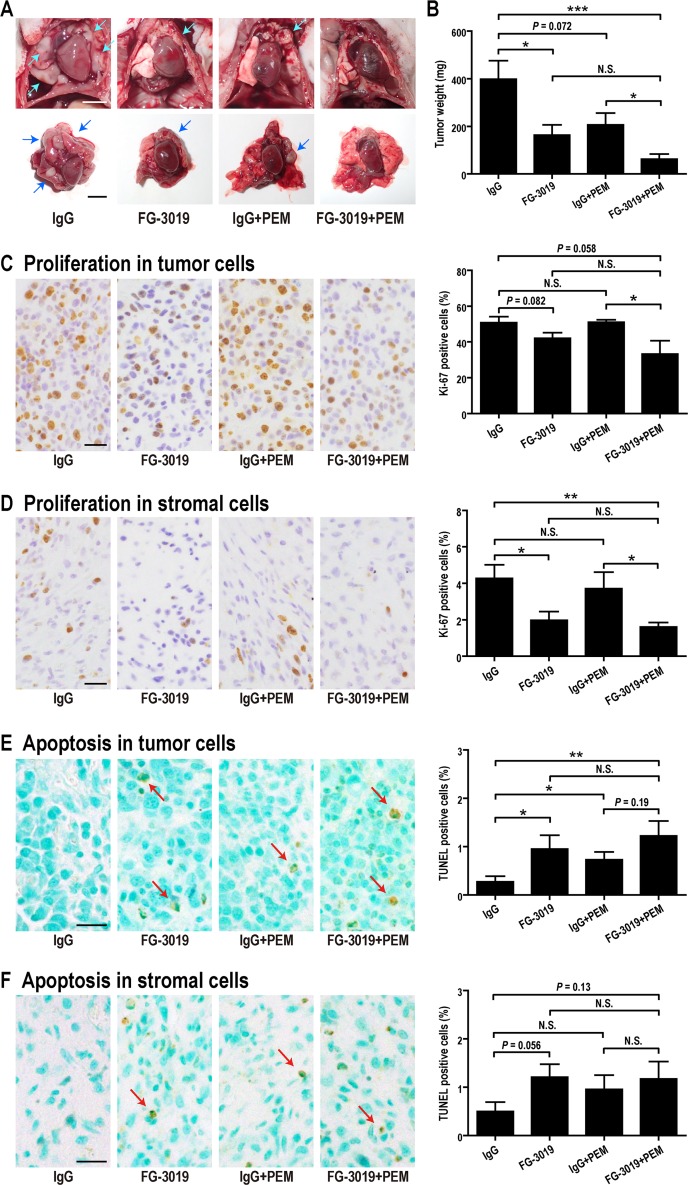
Effects of FG-3019 combined with PEM on the growth of orthotopic xenografts of a mesothelioma cell line, ACC-MESO-4 **(A)** Macroscopic findings. The tumors (arrows) grew in the pleural space (upper figure). The tumor including thoracic organs was dissected (lower figure). **(B)** Weight of tumors. Average tumor weights of FG-3019-treated group were significantly lower than IgG control group. **(C, D)** Quantitation of proliferation using Ki-67 antibody. **(E, F)** Quantitation of apoptosis using TUNEL assay (arrows). Color contrast was equally increased in (E) and (F). N = 6 (IgG), N = 6 (FG-3019), N = 6 (IgG + PEM), N = 6 (FG-3019 + PEM) in (B)–(F); means ± SEM, ^*^P < 0.05, ^**^P < 0.01, ^***^P < 0.005. N is the number of mice used. Pathological specimens were generated for each mouse and analyzed. Bar; 5 mm in (A), 20 μm in (C)-(F). N.S., not significant.

FG-3019 has shown inhibitory activity in preclinical animal models of pancreatic cancer, pre-B cell acute lymphoblastic leukemia and melanoma [23–25, 39]. In order to determine if FG-3019 exhibits activity in an *in vivo* model of mesothelioma, ACC-MESO-4 cells were implanted orthotopically (intrapleurally) into *BALB/c nu/nu* mice (Supplementary Figure 4A). A mouse in the IgG control group died of tumor progression at day 46, revealing the largest tumor in this experiment (725 mg). The remaining mice were euthanized at day 56-57 after intrapleural implantation of mesothelioma cells. At autopsy, tumors growing in the thoracic cavity were examined and dissected with heart, bilateral lungs and mediastinum (Figure 4A), followed by precise dissection of tumor tissue for weight measurement. In some mice, the tumor invaded the right thoracic cavity from the implantation site of the left thoracic cavity. There was a trend for the combination of FG-3019 + PEM to inhibit invasion into the opposite pleural cavity in comparison to IgG alone (*P* = 0.091, Supplementary Figure 4B). The average tumor weight was significantly lower in the FG-3019 group than in the IgG group (*P* < 0.05; Figure 4B). The average tumor weight was significantly lower in FG-3019 + PEM group than in the IgG group (*P* < 0.001) or in the IgG + PEM group (*P* < 0.05), demonstrating an enhanced effect.

To assess proliferation and apoptosis, immunohistochemical staining of Ki-67 and TUNEL assay were performed. It is not easy to distinguish fibroblasts and mesenchymal stem cells morphologically. In addition, CTGF can induce fibroblast differentiation [40] or differentiation of other cells [13, 20, 41] from mesenchymal stem cells. Therefore, we analyzed stromal cells, including fibroblasts and mesenchymal stem cells. Stromal cells were negative for calretinin and were spindle shaped. Blocking of CTGF with FG-3019 inhibited proliferation and induced apoptosis not only in tumor (mesothelioma) cells but also in stromal cells (*P* < 0.05; Figure 4C-4F). PEM significantly induced apoptosis in tumor cells (Figure 4E) but not in stromal cells (Figure 4F). The combinational effect of FG-3019 and PEM was apparent both in stromal cell proliferation (Figure 4D) and tumor cell apoptosis (Figure 4E), similar to that for tumor growth (Figure 4B).

**Figure 5:**
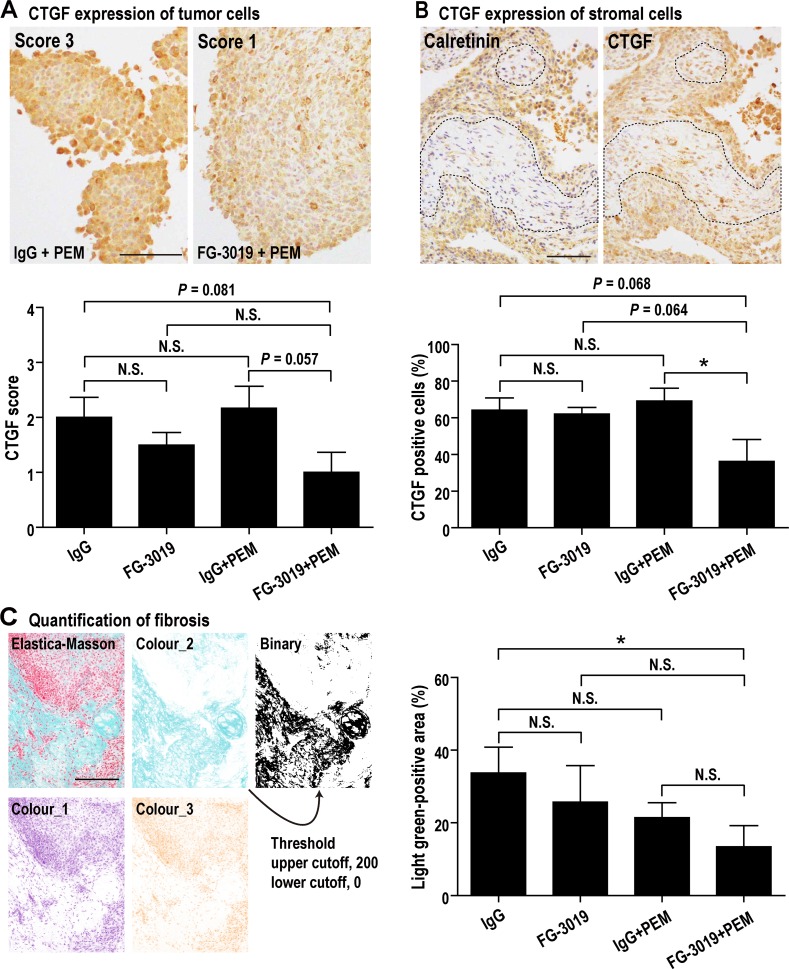
Effects of FG-3019 on CTGF expression and fibrosis **(A)** CTGF expression of tumor cells. Tumor (mesothelioma) cells were diffusely positive for CTGF. Degree of CTGF immunostaining in tumor cells (CTGF score) was evaluated as follows: 0, no staining or no mesothelioma cells; 1, weak; 2, moderate; 3, strong. CTGF score of FG-3019 + PEM group was lower than IgG + PEM group. **(B)** CTGF expression of stromal cells. For judging stromal portion in tumor (dotted line), we used H&E staining and immunohistochemical staining of calretinin (calretinin is a positive marker for mesothelioma). Stromal cells were negative for calretinin and spindle shaped (mesothelioma cells were cuboidal). Stromal cells were sporadically positive for CTGF, so we calculated the percentage of CTGF-positive stromal cells. The percentage of FG-3019 + PEM group was lower than the other group. **(C)** Tumor fibrosis (Elastica-Masson staining). Fibrosis, which is shown as light green positive, was quantified with ImageJ as described in Materials and Methods. FG-3019 + PEM significantly decreased the fibrosis of tumor. N = 6 (IgG), N = 6 (FG-3019), N = 6 (IgG + PEM), N = 6 (FG-3019 + PEM) in (A)–(C); means ± SEM, ^*^P < 0.05. N is the number of mice used. Pathological specimens generated for each mouse and analyzed. Bar; 50 μm in (A), 100 μm in (B), 200 μm in (C). N.S., not significant.

To evaluate CTGF expression and fibrosis, immunohistochemical staining of CTGF and Elastica-Masson staining were performed. Tumor cells were diffusely positive for CTGF whereas stromal cells were sporadically positive. The former was assessed by intensity of staining, and the latter was analyzed by percentage of positive cells. FG-3019 + PEM showed a tendency to suppress CTGF expression and decreased tumor-associated fibrosis (Figure 5A-5C). Vascular density was not significantly altered by FG-3019 and/or PEM (Supplementary Figure 4C).

### FG-3019 inhibits the expression of X-linked inhibitor of apoptosis (XIAP) protein in the xenograft model

**Figure 6:**
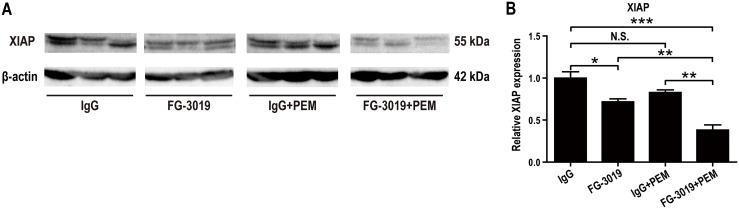
Mechanism of apoptosis induction by FG-3019 in mouse xenograft model **(A)** Western blot analysis of total lysates from grown mesothelioma tissue. **(B)** Corresponding densitometry of XIAP. Protein levels of XIAP were significantly decreased in FG-3019-treated group. There was a synergistic effect on the XIAP expression by the combination of FG-3019 + PEM. N = 3-4 for each group in (A), (B); means ± SEM, ^*^P < 0.05, ^**^P < 0.01, ^***^P < 0.005. Each protein level was normalized to β-actin. XIAP, X-linked inhibitor of apoptosis. N.S., not significant.

Caspase signaling is one of the important pathways regulating apoptosis. X-linked inhibitor of apoptosis (XIAP), which inhibits caspases, was reported to be suppressed in the lysates of FG-3019-treated murine pancreatic cancers and the effect was most pronounced in the FG-3019 + gemcitabine combination group [24]. Inhibitor of apoptosis (IAP) family proteins are also overexpressed in mesothelioma [42] and may regulate drug resistance [43] or pharmacological effects of chemotherapeutic drugs [43–45]. In western blot analysis, XIAP levels were significantly decreased in FG-3019-treated groups (*P* < 0.05; Figure 6A and 6B). This effect was strongest in the FG-3019 + PEM group, in which XIAP expression was significantly lower than that after monotherapy either with FG-3019 or PEM.

## DISCUSSION

In the present study, using an orthotopic mouse model, we demonstrated that FG-3019 attenuates mesothelioma growth by inhibiting proliferation and inducing apoptosis of both mesothelioma and stromal cells. Our data confirmed CTGF expression by primary fibroblasts, and showed that they promote proliferation and invasion of mesothelioma cells. Such effects have been recognized in various diseases, including other types of cancer [12–15], but this is the first report for mesothelioma. The observation that FG-3019 exhibited more significant anti-cancer effect *in vivo* than *in vitro* suggests that matricellular proteins like CTGF may require distinct microenvironmental factors or conditions that are difficult to be replicated in cell culture experiments.

It has been proposed that, not only tumor cells, but also the tumor microenvironment, including fibroblasts, vascular cells and inflammatory cells, are important for tumor progression [46, 47]. Although it remains controversial, targeting of stromal remodeling is being evaluated as a novel cancer therapy [48–50]. CTGF has been associated with renal fibrosis [12–14] and early clinical trials of FG-3019 were thus performed in subjects with diabetic nephropathy [51]. In animal cancer models, FG-3019 was reported to attenuate growth of PDAC [22–24], malignant melanoma [25] and ovarian cancer [26]. In our *in vitro* study, NHLF or rhCTGF promoted proliferation and migration/invasion of mesothelioma cells. This effect of rhCTGF was consistent with the effect of NHLF and with the results of previous studies on other models [23, 38]. Phase-2 clinical trials have been completed for IPF [27] and are in progress for PDAC [28]. Thus far, FG-3019 has exhibited no major side effects and has been well tolerated in IPF patients, with encouraging outcomes regarding pulmonary function and fibrosis [27]. Similarly, FG-3019 has been well tolerated in PDAC patients, and its combination with gemcitabine and erlotinib produced encouraging results [28].

Previous studies revealed that sarcomatoid-subtype mesothelioma expresses higher levels of CTGF than epithelioid-subtype by using tissue or serum samples [10, 11]. In the present study, however, all human mesothelioma cell lines expressed CTGF irrespective of histological subtype, and ACC-MESO-4 expressed especially high levels. This apparent inconsistency may be due to CTGF expression by stromal cells *in vivo*, although matrix stiffness in cell culture may also contribute to higher *in vitro* expression of mesothelial/mesothelioma cells. Sarcomatoid-subtype mesothelioma cells are commonly spindle shaped and accompanied by proliferation of non-neoplastic stromal cells, which makes it difficult to distinguish them.

It was reported that FG-3019 exhibited little effect on pancreatic cancer cell proliferation in conventional 2-dimensional cell culture *in vitro* whereas it strongly inhibited anchorage-independent growth [22]. ACC-MESO-4 appears to have low ability to proliferate in an anchorage-independent environment because it formed no colonies even in the IgG control group in soft agar colony formation assay. Similarly, when we transplanted ACC-MESO-4 without Matrigel matrix into left thoracic cavity in a preliminary experiment, the resulting tumor was too small to evaluate the therapeutic effects. Other investigators also reported that tumors were small when ACC-MESO-4 was transplanted without Matrigel matrix [34]. In our study, tumors grew large enough in the IgG control group to compare them with those of therapeutic groups because the Matrigel matrix could provide anchorage for tumor engraftment. In comparison to the control group, FG-3019 or FG-3019 + PEM attenuated tumor growth, and there were a few cases of complete growth inhibition. One interpretation of these results is that the mesothelioma cells did not engraft (not implanted efficiently) because FG-3019 prevented anchorage establishment by inhibiting CTGF. Such a mechanism has been reported for inhibition of ovarian cancer cells to disseminate in the peritoneum [26] and is consistent with our results of adhesion assay. For the tumors in which anchorage was established, FG-3019 inhibited proliferation and induced apoptosis both of mesothelioma cells and stromal cells, which probably resulted in a decreased tumor mass.

**Figure 7:**
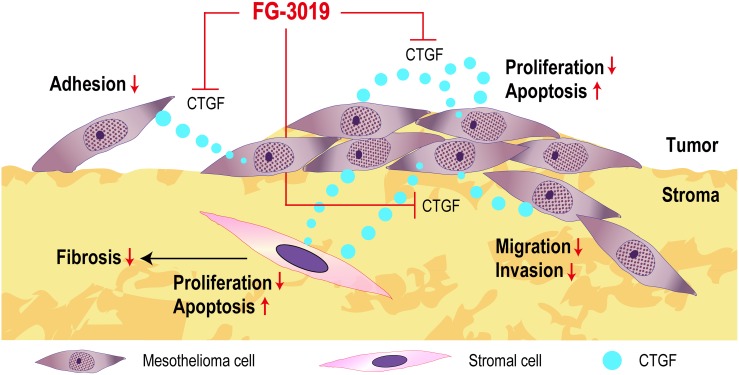
Summary scheme FG-3019 attenuated proliferation and induced apoptosis both in mesothelioma cells and stromal cells. FG-3019 may target both autocrine and paracrine CTGF effects on mesothelioma and stromal cells. Light blue circles indicate CTGF.

*CTGF* expression is under the regulation of TGF-β or Wnt signaling [14] and is secreted in an autocrine or paracrine manner [12, 13, 21]. In rat mesothelioma, an autocrine loop of CTGF promotes tumor growth [10]. The primary therapeutic benefit of FG-3019 would result from the inhibition of autocrine or paracrine CTGF effects (Figure 7). Combining drugs that act through different mechanisms would result in more effective inhibition of tumor growth. CTGF has been associated with tumor cell survival and drug resistance by inducing anti-apoptotic proteins, including Flip, Survivin, Bcl-xL and IAP [15, 52]. XIAP is known as a protein that inhibits the apoptotic pathway by inhibiting caspases, and its overexpression has been reported in human mesothelioma [42]. In a genetically engineered mouse model of PDAC, FG-3019 sensitized tumor cells to gemcitabine-induced apoptosis by inhibiting expression of XIAP [24]. Inhibition of BMP or of TGF-β receptors has been shown to downregulate the expression of *XIAP* [53]. Because CTGF has been reported to enhance TGF-β signals [12, 16], CTGF inhibition may lead to downregulation of XIAP in mesothelioma by altering these pathways. In our study, CTGF inhibition with FG-3019 induced apoptosis *in vivo*, and there were synergistic effects by the combination of FG-3019 + PEM. Western blot analysis also revealed that the combination of FG-3019 and PEM resulted in a greater decrease of *XIAP* expression than the monotherapy. Alternatively, CTGF has a proliferative role in cancers [15] and our study of Ki-67 immunostaining *in vivo* was consistent with this mechanistic hypothesis that CTGF block inhibits cell proliferation without alteration of vasculature density.

One limitation of the present study is that we adopted intraperitoneal injection of PEM because it is clinically administered to patients by intravenous injection and we felt that intrapleural injection of PEM would be too effective to evaluate the synergistic effects with FG-3019. On the other hand, we injected FG-3019 intrapleurally to directly act on mesothelioma because its effect was marginal *in vitro*. This route of administration could be performed for patients when pleural effusion is drained. Not much data are available regarding the effects of FG-3019 on the immune system, including programmed death-ligand 1, programmed death-1, antibody-dependent cell-mediated cytotoxicity and/or complement-dependent cytotoxicity. Further studies and discussions are necessary for the clinical study of FG-3019 on mesothelioma.

In conclusion, the human monoclonal antibody, FG-3019, was effective for mesothelioma in a murine orthotopic implant model, at least in a limited human mesothelioma cell line. Our results suggest that FG-3019 might be beneficial to human mesothelioma patients. There remains a strong medical need for more effective treatments of mesothelioma, and the data reported here provide a rationale to consider testing FG-3019 in clinical trials for mesothelioma patients as an addition to conventional therapies.

## MATERIALS AND METHODS

### Materials

We obtained FG-3019 and human IgG used as a control from FibroGen, Inc. (San Francisco, CA). FG-3019 is a fully human IgG_1_κ monoclonal antibody recognizing the *von Willebrand* factor module of human and rodent CTGF. PEM was purchased from Eli Lilly Japan (Kobe, Japan) and dissolved in sterile normal saline to 25 mg/ml. Bovine serum albumin (BSA), crystalized, was from Wako (Osaka, Japan).

### Cell lines and culture

MeT-5A, a human immortalized mesothelial cell line, was obtained from the American Type Culture Collection (ATCC; Manassas, VA) and maintained as described [44]. Six Japanese mesothelioma cell lines, ACC-MESO-1, ACC-MESO-4, Y-MESO-8A, Y-MESO-8D, Y-MESO-9 and Y-MESO-12, were established by Yoshitaka Sekido (Aichi Cancer Center Research Institute, Nagoya, Japan) [54, 55]. NCI-H290 and NCI-H2452 were kindly provided by Dr. Adi F. Gazdar (Hamon Center for Therapeutic Oncology, University of Texas Southwestern Medical Center, Dallas, TX). NCI-H2052, NCI-H2373 and MSTO-211H were purchased from ATCC. Normal human lung fibroblast (NHLF) was purchased from Lonza (Basel, Switzerland). The mesothelioma cell lines and NHLF were grown in RPMI 1640 medium, supplemented with 10% fetal bovine serum (FBS) and 1% antibiotic-antimycotic solution (Invitrogen, Carlsbad, CA). All the cell lines were cultured in a 5% CO_2_ humidified atmosphere at 37 °C and used at 10-20 passages.

### Western blot analysis

Whole cell lysates and homogenized tissue lysates were prepared in ice-cold Lysis-M buffer (Roche, Mannheim, Germany), supplemented with cOmplete Mini protease inhibitor and PhosSTOP phosphatase inhibitor tablets (Roche). The whole process after protein separation was performed as previously described [56]. We used 10% or 15% sodium dodecyl sulfate-polyacrylamide gel electrophoresis for protein separation. The primary antibodies and the dilutions used were: CTGF (Santa Cruz Biotechnology, Santa Cruz, CA; 14939, 1:200), X-linked inhibitor of apoptosis (Abcam, Cambridge, UK; 21278, 1:1,000), β-actin (Sigma-Aldrich, St. Louis; A1978, 1:1,000). Secondary antibodies were immunoglobulins conjugated to horseradish peroxidase (Dako, Glostrup, Denmark; 1:1,000). Immunodetection was performed using Amersham ECL Western Blotting Detection Reagent (GE Healthcare, Little Chalfont, Buckinghamshire, UK). Densitometric analysis was performed using the ImageJ software (http://rsb.info.nih.gov/ij/).

### MTT assay

Cells (2-10×10^3^ per well; the same number of cells for each cell line) were seeded in a 96-well plate and MTT assay was performed using the method described previously [57]. For dose-response analyses, PEM concentration was 0.1, 0.5, 1, 5 and 10 μM. For time-course analyses, the concentrations of IgG/FG-3019 and PEM were 100 μg/ml and 1 μM, respectively.

### TUNEL assay

Cells (1-2×10^5^ per well; the same number of cells for each cell line) were seeded in a 6-well plate and incubated for 24 h. The supernatant was then discarded and the medium containing the above-mentioned drugs (IgG/FG-3019: 100 μg/ml, PEM: 1 μM; final concentration) was added. Cells were collected 48 h after addition of agents and fixed with PBS-buffered 10% formalin solution (Wako). TUNEL assay was performed using the apoptosis *in situ* detection kit (Wako) according to the manufacturer's protocol. Apoptosis was quantified by counting more than 400 cells in more than four different fields on each filter, using a light microscope at 200 × magnification.

### Migration and invasion assays

Cell migration and invasion assays were performed using transwell permeable supports (Corning Incorporated, Life Science, New York, NY) with 8 μm-pore filters in the same manner as described [57]. We used 3 μl of Corning Matrigel matrix (Corning, typical protein concentration: 9-12 mg/ml) for coating each upper chamber for invasion according to the manufacturer's protocol. BSA was dissolved to 0.1% in serum-free medium. Cells (ACC-MESO-4, 4×10^4^; Y-MESO-8D, 1×10^4^ and NCI-H290, 2×10^4^ each well) were seeded into the upper chambers with 0.1% BSA medium, and medium with 10% FBS and the above-mentioned drugs (IgG/FG-3019: 100 μg/ml, PEM: 1 μM; final concentration) were placed in the lower chambers. Hematoxylin was used for staining the cells. Migration and invasion were quantified by counting cells in five different fields on each filter under a light microscope at 100 × magnification.

### Adhesion assay

A 96-well plate was coated with 100 μl of Matrigel matrix (Corning, final protein concentration: 9-12 μg/ml) in PBS overnight at 4 °C. Thereafter, the plate was washed three times with PBS, blocked with PBS containing 1% BSA for 1 h at 37 °C, and rewashed with PBS. Cells were resuspended in 0.1% BSA medium (serum-free) with the drugs (IgG/FG-3019: 100 μg/ml, PEM: 1 μM; final concentration), placed at a density of 2×10^4^ per well, and incubated for 1 h. After removing unattached cells by washing three times with serum-free medium, we performed MTT assay to measure the attached cells.

### Soft agar colony formation assay

Cells (ACC-MESO-4 5×10^5^, Y-MESO-8D 2×10^5^, NCI-H290 2×10^5^) were resuspended in 1.5 ml medium containing 0.36% Noble Agar (Becton Dickinson and Company, Franklin Lakes, NJ), 10% FBS and the above-mentioned drugs (IgG/FG-3019: 100 μg/ml, PEM: 1 μM; final concentration). Each cell mixture was overlaid on 2.5 ml medium containing 0.75% Noble Agar containing 10% FBS in a 6-well plate. The plate was incubated in a humidified incubator at 37 °C for 30 days and the colonies were counted in 5 fields on each well using a light microscope at 100 × magnification.

### Co-culture experiments of mesothelioma cells and fibroblasts

To assess the effects of NHLF on mesothelioma cell proliferation, NHLF (1×10^4^ each chamber) were seeded into the upper chamber (0.4 μm filters, Corning) and mesothelioma cells (1×10^4^ each chamber) were plated in the lower chamber of transwell systems. After incubation of 3, 5 or 7 days, cells from each chamber were collected and the cell suspension was counted with a standard hemocytometer. Using the same method, mesothelioma cells (1×10^5^ per chamber) and NHLF (1×10^5^ per chamber) were seeded and separately collected after 48 h-incubation and used to prepare lysates for western blot analysis. Relative CTGF expression in comparison to each mono-culture experiment was calculated after semiquantitative analysis with ImageJ.

We performed migration and invasion assays in the same way mentioned above. Mesothelioma cells (1×10^4^ per chamber) were seeded into the upper chamber, and NHLF (1×10^4^ per chamber) were plated in the lower chamber of transwell systems. To assess the effect of NHLF, mesothelioma cells were cultured with or without NHLF. To analyze the effect of drugs on the migration/invasion behavior of mesothelioma cells in circumstances similar to *in vivo*, migration/invasion assays were performed in co-culture conditions.

### rhCTGF experiments on mesothelioma cells

rhCTGF (RD172035100-HEK) was obtained from BioVendor Laboratory Medicine, Inc. (Heidelberg, Germany). Based on previous studies [23, 38], cells (5×10^3^ per well) were incubated in a 96-well plate with or without rhCTGF (100 or 200 ng/ml) for 24 h, and MTT assay was performed. Regarding migration and invasion assays, cells (1×10^4^ per chamber) were seeded into the upper chambers with 0.1% BSA medium, and 1% FBS medium with or without rhCTGF (200 ng/ml) was placed in the lower chambers.

### Orthotopic xenograft experiment

Male *BALB/c nu/nu* mice, 4 weeks of age, were obtained from SLC (Hamamatsu, Japan). ACC-MESO-4 (2×10^6^ cells) were resuspended in 50 μl of RPMI 1640 (FBS free), and 50 μl of Corning Matrigel matrix (Corning) was added to this suspension. The mice were transplanted into the left pleural cavity with 100 μl of the cell suspension using a 26-gauge needle. Mice were divided into four groups; IgG (N = 6), FG-3019 (N = 6), IgG + PEM (N = 6) and FG-3019 + PEM (N = 6). IgG or FG-3019 was administered at 40 mg/kg intrapleurally twice a week (firstly 24 h after mesothelioma transplantation); PEM was administered at 50 mg/kg intraperitoneally once a week. These drug therapies were performed from 1 to 8 weeks after mesothelioma transplantation (day 1-55). At 9 weeks after transplantation (day 56-57), the mice were euthanized and the pleural cavity was examined. Thoracic components, including organs and tumor, were dissected. Tumor weight was measured precisely after removing heart, lungs, trachea, esophagus, mediastinal adipose tissue and diaphragm. Half of the tumor was fixed in PBS-buffered 10% formalin solution (Wako) for histology, and the other half was stored at −80 °C for subsequent analyses. All the procedures were performed in accordance with the national guidelines and approved by the animal experiment committee of Nagoya University Graduate School of Medicine.

### Histologic and immunohistochemical analysis

Pathological specimens were generated for each mouse after autopsy. Tissues were fixed in neutral buffered 10% formalin solution for 24 h and then washed with 70% ethanol. Tissues were embedded in paraffin and 4 μm sections were stained either with hematoxylin and eosin (H&E), Elastica-Masson or immunohistochemistry. Immunohistochemical analysis was performed with the avidin-biotin complex method using peroxidase, as previously described [58] The following antibodies and kits were used for immunohistochemistry: CTGF (Santa Cruz Biotechnology, #14939; 1:200), calretinin (Abcam, #16694; 1:100), Ki-67, clone SP6 (Abcam, #16667; 1:100), TUNEL (Wako), and CD31 (Abcam, #28364, 1:50). Antigen retrieval for Ki-67 was performed by heating the sections with microwave in 10 mM citrate buffer, pH 6.0. For CTGF, calretinin and CD31, we used 10 mM Tris (hydroxymethyl) aminomethane/1 mM ethylenediaminetetraacetic acid buffer, pH 9.0. Tissue sections were blocked with Protein Block Serum-Free Ready-to-use (Dako). Ki-67 staining and TUNEL assay were assessed by counting 200-1000 cells in the hot spot areas (most frequently positive areas). Vascular density was assessed over the whole area of each tumor. All images were obtained using an Olympus microscope BX53 (Tokyo, Japan; objective lense; UPLSAPO) and a camera DP22/U-TV0.5XC. CTGF immunostaining in tumor cells (CTGF score) was semiquantitated as follows: 0, no staining or no mesothelioma cells; 1, weak; 2, moderate; 3, strong. We calculated the percentage of CTGF-positive stromal cells in the hot spot area. Two registered pathologists (K.Y. and S.T.) and one resident pathologist (Y.O.) assessed these histologic/immunohistochemical analyses.

### Semi-quantitative imaging analysis of fibrosis in tumor

Fibrosis was detected by light green in Elastica-Masson staining. All xenograft histological specimens stained by light green were digitized, using the microscope and camera described above. We analyzed the image using the ImageJ software and color deconvolution plugin (http://imagej.net/Colour_Deconvolution) for ImageJ, and Fiji implements staining separation by the method of Ruifrok and Johnston [59]. Extracted light green-positive area was converted to black (threshold: upper cutoff, 200; lower cutoff, 0) with reduction of noises. After these processes, most of the remaining dots in the image were those originally stained in light green, and we thereafter measured the whole area of the dots. This area was divided by the entire tumor mass area and the percentage of fibrosis was obtained.

### Statistical analysis

The data were analyzed using SPSS Statistics 24.0 (IBM, Armonk, NY). Statistical significance between two groups of interest was analyzed using the unpaired Student's *t*-test if not indicated otherwise. The results were shown as the mean ± SEM except for where noted. A *P* value of < 0.05 was considered significant.

## SUPPLEMENTARY MATERIALS FIGURES AND TABLES



## Abbreviations

BSA: bovine serum albumin
BMP: bone morphogenetic protein
CDDP: cisplatin
CTGF: connective tissue growth factor
FBS: fetal bovine serum
IAP: inhibitor of apoptosis
IPF: idiopathic pulmonary fibrosis
LRP: low density lipoprotein receptor-related protein
MTT: 4,5-*di*-methylthiazolyl-2-*yl*)-2,5-diphenyltetrazolium bromide;
NHLF: normal human lung fibroblast
PBS: phosphate-buffered saline
PDAC: pancreatic ductal adenocarcinoma
PEM: pemetrexed
TGF-β: transforming growth factor-β
TUNEL: terminal deoxynucleotidyl transferase dUTP nick end labeling
XIAP: X-linked inhibitor of apoptosis.

## References

[1] Oury TD, Roggli VL, Sporn TA (2014). Pathology of asbestos-associated diseases.

[2] IARC, WHO (2012). Asbestos (chrysotile, amosite, crocidolite, tremolite, actinolite, and anthophyllite). IARC monographs on the evaluation of carcinogenic risks to humans. a review of human carcinogens; part c: Arsenic, metals, fibres, and dusts.

[3] Robinson BW, Lake RA (2005). Advances in malignant mesothelioma. N Engl J Med.

[4] Hodgson JT, McElvenny DM, Darnton AJ, Price MJ, Peto J (2005). The expected burden of mesothelioma mortality in Great Britain from 2002 to 2050. Br J Cancer.

[5] Inui T, Yasutaka T, Endo K, Katsumi T (2012). Geo-environmental issues induced by the 2011 off the pacific coast of Tohoku earthquake and tsunami. Soils Found.

[6] Offenberg JH, Eisenreich SJ, Chen LC, Cohen MD, Chee G, Prophete C, Weisel C, Lioy PJ (2003). Persistent organic pollutants in the dusts that settled across lower Manhattan after September 11, 2001. Environ Sci Technol.

[7] Rusch VW, Giroux D, Kennedy C, Ruffini E, Cangir AK, Rice D, Pass H, Asamura H, Waller D, Edwards J, Weder W, Hoffmann H, van Meerbeeck JP (2012). Initial analysis of the international association for the study of lung cancer mesothelioma database. J Thorac Oncol.

[8] Toyokuni S (2009). Role of iron in carcinogenesis: cancer as a ferrotoxic disease. Cancer Sci.

[9] Ohara Y, Chew SH, Shibata T, Okazaki Y, Yamashita K, Toyokuni S (2018). Phlebotomy as a preventive measure for crocidolite-induced mesothelioma in male rats. Cancer Sci.

[10] Jiang L, Yamashita Y, Chew SH, Akatsuka S, Ukai S, Wang S, Nagai H, Okazaki Y, Takahashi T, Toyokuni S (2014). Connective tissue growth factor and beta-catenin constitute an autocrine loop for activation in rat sarcomatoid mesothelioma. J Pathol.

[11] Fujii M, Toyoda T, Nakanishi H, Yatabe Y, Sato A, Matsudaira Y, Ito H, Murakami H, Kondo Y, Kondo E, Hida T, Tsujimura T, Osada H (2012). TGF-beta synergizes with defects in the hippo pathway to stimulate human malignant mesothelioma growth. J Exp Med.

[12] Gressner OA, Gressner AM (2008). Connective tissue growth factor: a fibrogenic master switch in fibrotic liver diseases. Liver Int.

[13] Kubota S, Takigawa M (2015). Cellular and molecular actions of CCN2/CTGF and its role under physiological and pathological conditions. Clin Sci (Lond).

[14] Chu CY, Chang CC, Prakash E, Kuo ML (2008). Connective tissue growth factor (CTGF) and cancer progression. J Biomed Sci.

[15] Wells JE, Howlett M, Cole CH, Kees UR (2015). Deregulated expression of connective tissue growth factor (CTGF/CCN2) is linked to poor outcome in human cancer. Int J Cancer.

[16] Abreu JG, Ketpura NI, Reversade B, De Robertis EM (2002). Connective-tissue growth factor (CTGF) modulates cell signalling by BMP and TGF-beta. Nat Cell Biol.

[17] Liu SC, Chuang SM, Hsu CJ, Tsai CH, Wang SW, Tang CH (2014). CTGF increases vascular endothelial growth factor-dependent angiogenesis in human synovial fibroblasts by increasing miR-210 expression. Cell Death Dis.

[18] Shimo T, Nakanishi T, Nishida T, Asano M, Kanyama M, Kuboki T, Tamatani T, Tezuka K, Takemura M, Matsumura T, Takigawa M (1999). Connective tissue growth factor induces the proliferation, migration, and tube formation of vascular endothelial cells *in vitro*, and angiogenesis *in vivo*. J Biochem.

[19] Nishida T, Kondo S, Maeda A, Kubota S, Lyons KM, Takigawa M (2009). CCN family 2/connective tissue growth factor (CCN2/CTGF) regulates the expression of VEGF through hif-1alpha expression in a chondrocytic cell line, hcs-2/8, under hypoxic condition. Bone.

[20] Luo Q, Kang Q, Si W, Jiang W, Park JK, Peng Y, Li X, Luu HH, Luo J, Montag AG, Haydon RC, He TC (2004). Connective tissue growth factor (CTGF) is regulated by wnt and bone morphogenetic proteins signaling in osteoblast differentiation of mesenchymal stem cells. J Biol Chem.

[21] Yang J, Velikoff M, Canalis E, Horowitz JC, Kim KK (2014). Activated alveolar epithelial cells initiate fibrosis through autocrine and paracrine secretion of connective tissue growth factor. Am J Physiol Lung Cell Mol Physiol.

[22] Dornhofer N, Spong S, Bennewith K, Salim A, Klaus S, Kambham N, Wong C, Kaper F, Sutphin P, Nacamuli R, Hockel M, Le Q, Longaker M (2006). Connective tissue growth factor-specific monoclonal antibody therapy inhibits pancreatic tumor growth and metastasis. Cancer Res.

[23] Aikawa T, Gunn J, Spong SM, Klaus SJ, Korc M (2006). Connective tissue growth factor-specific antibody attenuates tumor growth, metastasis, and angiogenesis in an orthotopic mouse model of pancreatic cancer. Mol Cancer Ther.

[24] Neesse A, Frese KK, Bapiro TE, Nakagawa T, Sternlicht MD, Seeley TW, Pilarsky C, Jodrell DI, Spong SM, Tuveson DA (2013). CTGF antagonism with mAb FG-3019 enhances chemotherapy response without increasing drug delivery in murine ductal pancreas cancer. Proc Natl Acad Sci U S A.

[25] Finger EC, Cheng CF, Williams TR, Rankin EB, Bedogni B, Tachiki L, Spong S, Giaccia AJ, Powell MB (2014). CTGF is a therapeutic target for metastatic melanoma. Oncogene.

[26] Moran-Jones K, Gloss BS, Murali R, Chang DK, Colvin EK, Jones MD, Yuen S, Howell VM, Brown LM, Wong CW, Spong SM, Scarlett CJ, Hacker NF (2015). Connective tissue growth factor as a novel therapeutic target in high grade serous ovarian cancer. Oncotarget.

[27] Raghu G, Scholand MB, de Andrade J, Lancaster L, Mageto Y, Goldin J, Brown KK, Flaherty KR, Wencel M, Wanger J, Neff T, Valone F, Stauffer J (2016). FG-3019 anti-connective tissue growth factor monoclonal antibody: results of an open-label clinical trial in idiopathic pulmonary fibrosis. Eur Respir J.

[28] Picozzi VJ, Pipas JM, Koong AC, Giaccia AJ, Bahary N, Krishnamurthi SS, Lopez CD (2017). FG-3019, a human monoclonal antibody to connective tissue growth factor, combined with chemotherapy in patients with locally advanced or metastatic pancreatic ductal adenocarcinoma. J Cancer Clin Trials.

[29] Scherpereel A, Astoul P, Baas P, Berghmans T, Clayson H, de Vuyst P, Dienemann H, Galateau-Salle F, Hennequin C, Hillerdal G, Le Pechoux C, Mutti L, Pairon JC (2010). Guidelines of the european respiratory society and the european society of thoracic surgeons for the management of malignant pleural mesothelioma. Eur Respir J.

[30] Vogelzang NJ, Rusthoven JJ, Symanowski J, Denham C, Kaukel E, Ruffie P, Gatzemeier U, Boyer M, Emri S, Manegold C, Niyikiza C, Paoletti P (2003). Phase III study of pemetrexed in combination with cisplatin versus cisplatin alone in patients with malignant pleural mesothelioma. J Clin Oncol.

[31] Hanna N, Shepherd FA, Fossella FV, Pereira JR, De Marinis F, von Pawel J, Gatzemeier U, Tsao TC, Pless M, Muller T, Lim HL, Desch C, Szondy K (2004). Randomized phase III trial of pemetrexed versus docetaxel in patients with non-small-cell lung cancer previously treated with chemotherapy. J Clin Oncol.

[32] Taylor P, Castagneto B, Dark G, Marangolo M, Scagliotti GV, van Klaveren RJ, Labianca R, Serke M, Schuette W, van Meerbeeck JP, Heigener D, Liu Y, Adachi S (2008). Single-agent pemetrexed for chemonaive and pretreated patients with malignant pleural mesothelioma: results of an international expanded access program. J Thorac Oncol.

[33] Latz JE, Chaudhary A, Ghosh A, Johnson RD (2006). Population pharmacokinetic analysis of ten phase II clinical trials of pemetrexed in cancer patients. Cancer Chemother Pharmacol.

[34] Iwahori K, Serada S, Fujimoto M, Ripley B, Nomura S, Mizuguchi H, Shimada K, Takahashi T, Kawase I, Kishimoto T, Naka T (2013). SOCS-1 gene delivery cooperates with cisplatin plus pemetrexed to exhibit preclinical antitumor activity against malignant pleural mesothelioma. Int J Cancer.

[35] Hanauske AR, Eismann U, Oberschmidt O, Pospisil H, Hoffmann S, Hanauske-Abel H, Ma D, Chen V, Paoletti P, Niyikiza C (2007). *In vitro* chemosensitivity of freshly explanted tumor cells to pemetrexed is correlated with target gene expression. Invest New Drugs.

[36] Kawabata S, Chiang CT, Tsurutani J, Shiga H, Arwood ML, Komiya T, Gills JJ, Memmott RM, Dennis PA (2014). Rapamycin downregulates thymidylate synthase and potentiates the activity of pemetrexed in non-small cell lung cancer. Oncotarget.

[37] Nutt JE, Razak AR, O’Toole K, Black F, Quinn AE, Calvert AH, Plummer ER, Lunec J (2010). The role of folate receptor alpha (FRalpha) in the response of malignant pleural mesothelioma to pemetrexed-containing chemotherapy. Br J Cancer.

[38] Kawaki H, Kubota S, Suzuki A, Yamada T, Matsumura T, Mandai T, Yao M, Maeda T, Lyons KM, Takigawa M (2008). Functional requirement of CCN2 for intramembranous bone formation in embryonic mice. Biochem Biophys Res Commun.

[39] Lu H, Kojima K, Battula VL, Korchin B, Shi Y, Chen Y, Spong S, Thomas DA, Kantarjian H, Lock RB, Andreeff M, Konopleva M (2014). Targeting connective tissue growth factor (CTGF) in acute lymphoblastic leukemia preclinical models: anti-CTGF monoclonal antibody attenuates leukemia growth. Ann Hematol.

[40] Lee CH, Shah B, Moioli EK, Mao JJ (2010). CTGF directs fibroblast differentiation from human mesenchymal stem/stromal cells and defines connective tissue healing in a rodent injury model. J Clin Invest.

[41] Wagner W, Wein F, Seckinger A, Frankhauser M, Wirkner U, Krause U, Blake J, Schwager C, Eckstein V, Ansorge W, Ho AD (2005). Comparative characteristics of mesenchymal stem cells from human bone marrow, adipose tissue, and umbilical cord blood. Exp Hematol.

[42] Kleinberg L, Lie AK, Florenes VA, Nesland JM, Davidson B (2007). Expression of inhibitor-of-apoptosis protein family members in malignant mesothelioma. Hum Pathol.

[43] Gordon GJ, Mani M, Mukhopadhyay L, Dong L, Yeap BY, Sugarbaker DJ, Bueno R (2007). Inhibitor of apoptosis proteins are regulated by tumour necrosis factor-alpha in malignant pleural mesothelioma. J Pathol.

[44] Cregan IL, Dharmarajan AM, Fox SA (2013). Mechanisms of cisplatin-induced cell death in malignant mesothelioma cells: role of inhibitor of apoptosis proteins (IAPs) and caspases. Int J Oncol.

[45] Gordon GJ, Mani M, Maulik G, Mukhopadhyay L, Yeap BY, Kindler HL, Salgia R, Sugarbaker DJ, Bueno R (2008). Preclinical studies of the proteasome inhibitor bortezomib in malignant pleural mesothelioma. Cancer Chemother Pharmacol.

[46] Hanahan D, Weinberg RA (2011). Hallmarks of cancer: the next generation. Cell.

[47] Orimo A, Weinberg RA (2006). Stromal fibroblasts in cancer: a novel tumor-promoting cell type. Cell Cycle.

[48] Hingorani SR, Harris WP, Beck JT, Berdov BA, Wagner SA, Pshevlotsky EM, Tjulandin SA, Gladkov OA, Holcombe RF, Korn R, Raghunand N, Dychter S, Jiang P (2016). Phase ib study of PEGylated recombinant human hyaluronidase and gemcitabine in patients with advanced pancreatic cancer. Clin Cancer Res.

[49] Koliaraki V, Pasparakis M, Kollias G (2015). IKKbeta in intestinal mesenchymal cells promotes initiation of colitis-associated cancer. J Exp Med.

[50] Pallangyo CK, Ziegler PK, Greten FR (2015). IKKbeta acts as a tumor suppressor in cancer-associated fibroblasts during intestinal tumorigenesis. J Exp Med.

[51] Adler SG, Schwartz S, Williams ME, Arauz-Pacheco C, Bolton WK, Lee T, Li D, Neff TB, Urquilla PR, Sewell KL (2010). Phase 1 study of anti-CTGF monoclonal antibody in patients with diabetes and microalbuminuria. Clin J Am Soc Nephrol.

[52] Wang MY, Chen PS, Prakash E, Hsu HC, Huang HY, Lin MT, Chang KJ, Kuo ML (2009). Connective tissue growth factor confers drug resistance in breast cancer through concomitant up-regulation of BCL-Xl and CIAP1. Cancer Res.

[53] Augeri DJ, Langenfeld E, Castle M, Gilleran JA, Langenfeld J (2016). Inhibition of BMP and of TGFbeta receptors downregulates expression of XIAP and TAK1 leading to lung cancer cell death. Mol Cancer.

[54] Usami N, Fukui T, Kondo M, Taniguchi T, Yokoyama T, Mori S, Yokoi K, Horio Y, Shimokata K, Sekido Y, Hida T (2006). Establishment and characterization of four malignant pleural mesothelioma cell lines from Japanese patients. Cancer Sci.

[55] Tanaka I, Osada H, Fujii M, Fukatsu A, Hida T, Horio Y, Kondo Y, Sato A, Hasegawa Y, Tsujimura T, Sekido Y (2015). Lim-domain protein AJUBA suppresses malignant mesothelioma cell proliferation via hippo signaling cascade. Oncogene.

[56] Chew SH, Okazaki Y, Akatsuka S, Wang S, Jiang L, Ohara Y, Ito F, Saya H, Sekido Y, Toyokuni S (2017). Rheostatic CD44 isoform expression and its association with oxidative stress in human malignant mesothelioma. Free Radic Biol Med.

[57] Wang S, Jiang L, Han Y, Chew SH, Ohara Y, Akatsuka S, Weng L, Kawaguchi K, Fukui T, Sekido Y, Yokoi K, Toyokuni S (2016). Urokinase-type plasminogen activator receptor promotes proliferation and invasion with reduced cisplatin sensitivity in malignant mesothelioma. Oncotarget.

[58] Chew SH, Okazaki Y, Nagai H, Misawa N, Akatsuka S, Yamashita K, Jiang L, Yamashita Y, Noguchi M, Hosoda K, Sekido Y, Takahashi T, Toyokuni S (2014). Cancer-promoting role of adipocytes in asbestos-induced mesothelial carcinogenesis through dysregulated adipocytokine production. Carcinogenesis.

[59] Ruifrok AC, Johnston DA (2001). Quantification of histochemical staining by color deconvolution. Anal Quant Cytol Histol.

