# Supplementary material for: Connective tissue growth factor-specific monoclonal antibody inhibits growth of malignant mesothelioma in an orthotopic mouse model
Source: Oncotarget. 2018 Apr 6;9(26):18494–509. doi: 10.18632/oncotarget.24892 (PMC5915087; doi:10.18632/oncotarget.24892)
Supplement: Supplementary file 1 [file oncotarget-09-18494-s001.pdf]

Connective tissue growth factor-specific monoclonal antibody inhibits growth of malignant mesothelioma in an orthotopic mouse model

SUPPLEMENTARY MATERIALS

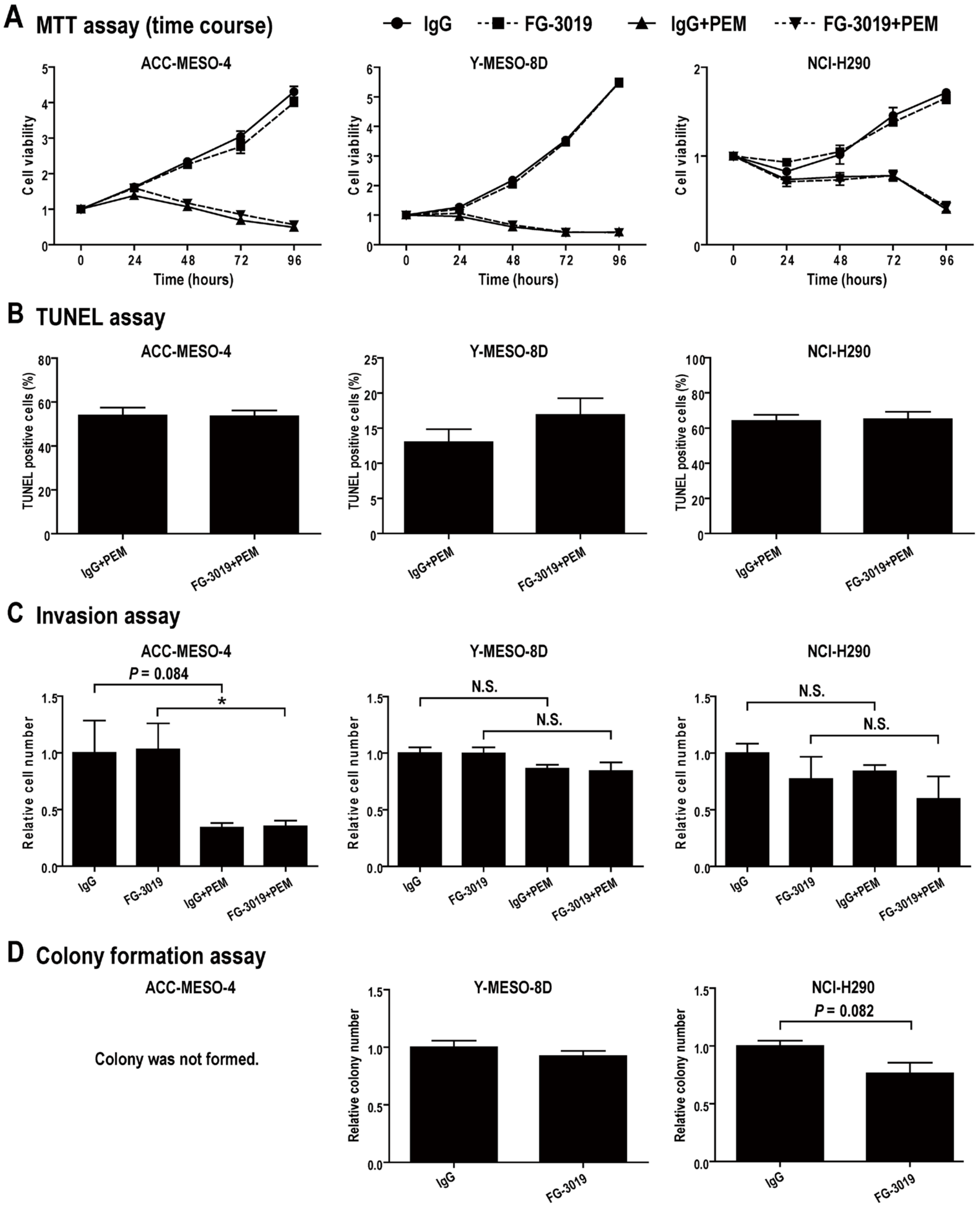

Supplementary Figure 1: Effects of FG-3019 on MM cell lines (ACC-MESO-4, Y-MESO-8D and NCI-H290) *in vitro*. **(A)** Effect of FG-3019 and PEM on cell viability. MTT assay was performed after 24-96 h-incubation in the presence of both drugs. **(B)** TUNEL assay. **(C)** Invasion assay. **(D)** Soft agar colony formation assay. Colony number was counted after 30-day incubation in agar with drugs. FG-3019 marginally inhibited the anchorage-independent growth only in NCI-H290 ($P = 0.082$). Colony was not formed in ACC-MESO-4 and in the other two cell lines in the presence of PEM. N = 3 for each group; means ± SEM. IgG/FG-3019: 100 μg/ml, PEM: 1 μM. PEM, pemetrexed; N.S., not significant.

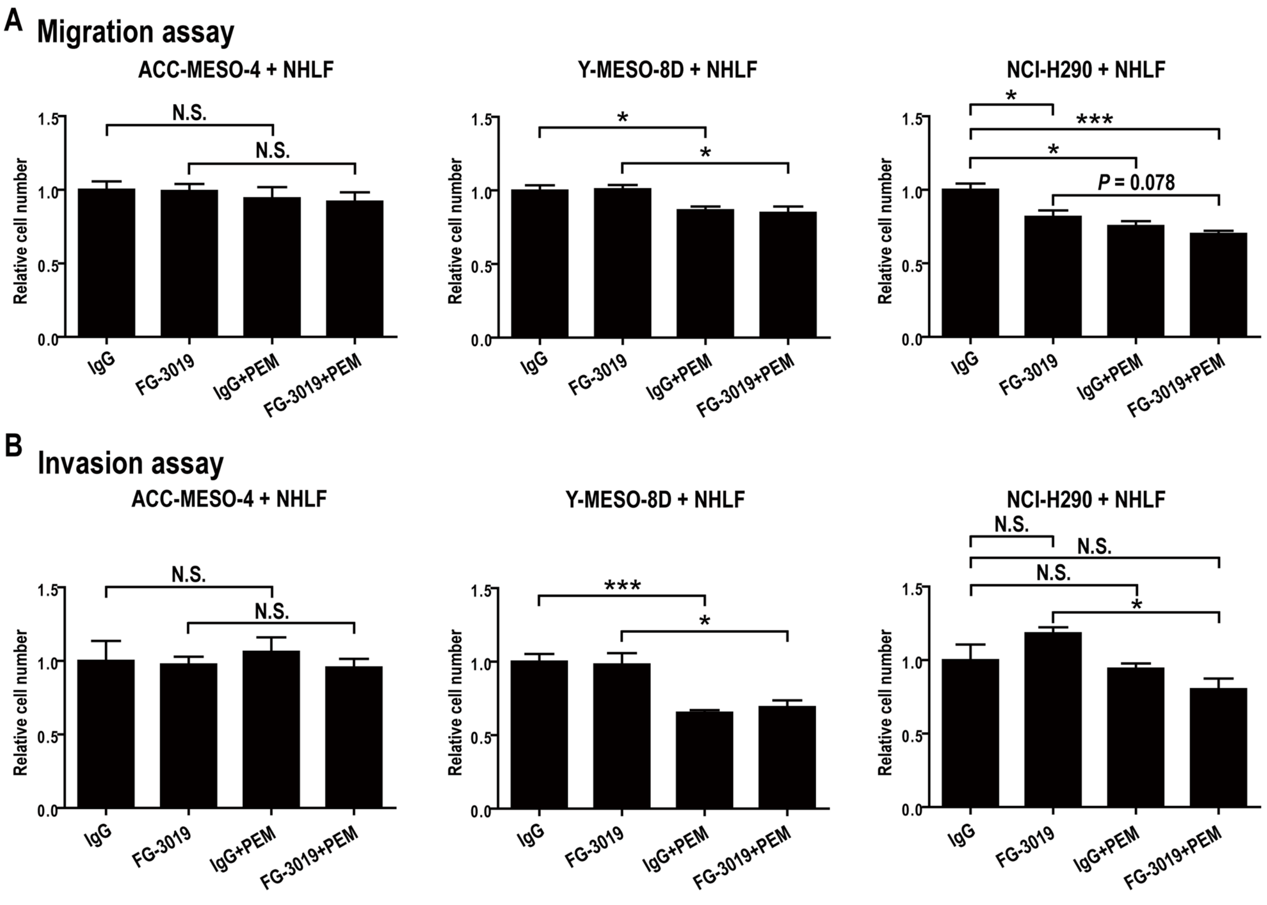

Supplementary Figure 2: Migration and invasion assay in co-culture with NHLF. **(A)** Migration assay. **(B)** Invasion assay. Only in migration assay on co-culture of NCI-H290 with NHLF, FG-3019 was effective. However, co-culture had no significant impact on the lack of response to FG-3019 in the other experiments. N = 3 for each group in (A) and (B); means ± SEM, $^{*}P < 0.05$, $^{**}P < 0.01$, $^{***}P < 0.005$. NHLF, normal human lung fibroblast; N.S., not significant.

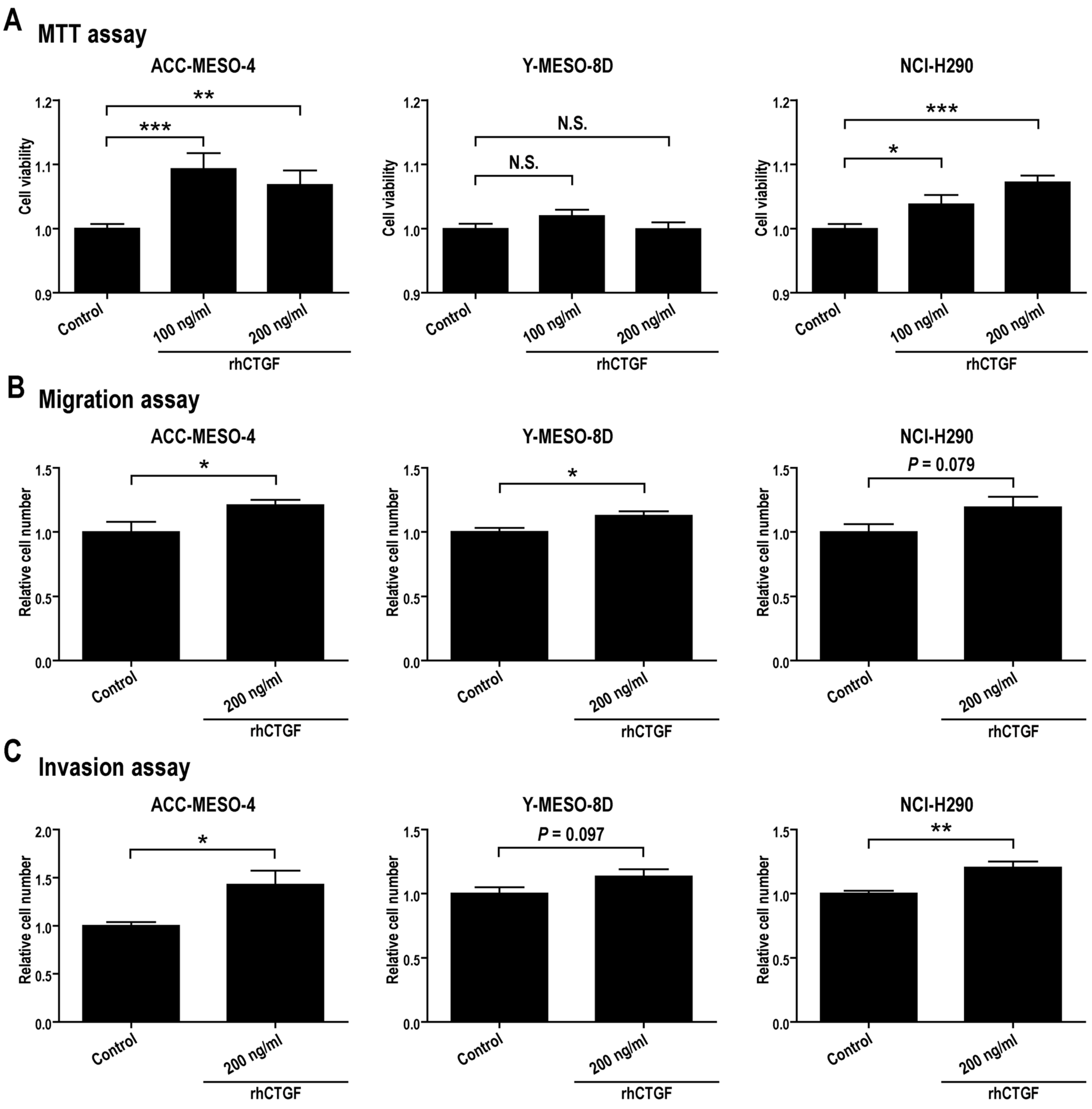

Supplementary Figure 3: Effects of rhCTGF on mesothelioma cell lines. **(A)** Effect of rhCTGF on cell viability. rhCTGF promoted proliferation of ACC-MESO-4 and NCI-H290. **(B, C)** Migration/Invasion assay. rhCTGF promoted migration and invasion of three mesothelioma cell lines. N = 3 for each group in (A, C); means ± SEM, $^{*}P < 0.05$, $^{**}P < 0.01$, $^{***}P < 0.005$. rhCTGF, recombinant human CTGF.

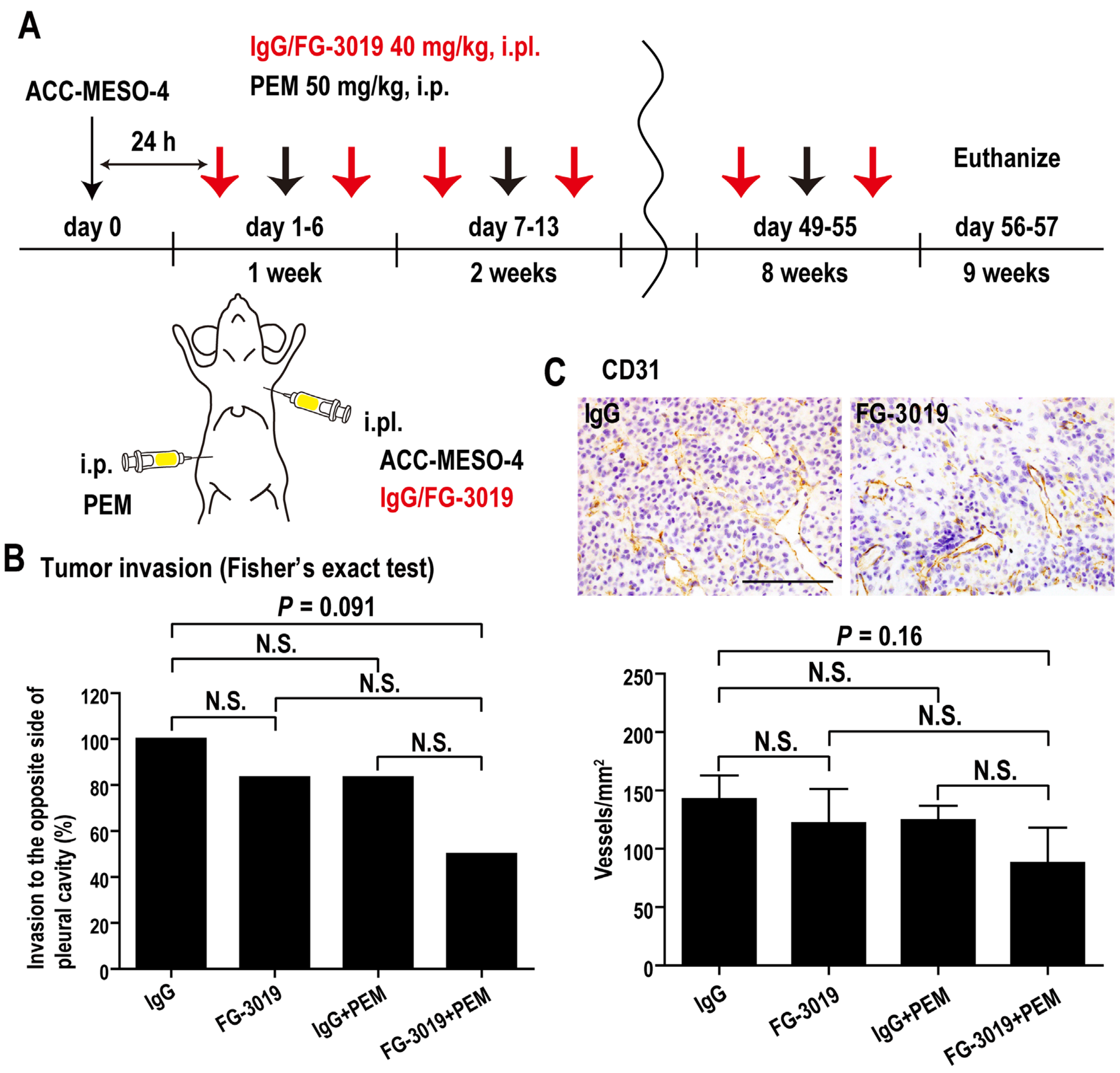

Supplementary Figure 4: Macroscopic and microscopic findings of *in vivo* model. (A) Schematic of the *in vivo* experiment for the combination therapy of FG-3019 and PEM. **(B)** Invasion to the opposite side of thoracic cavity. FG-3019 + PEM marginally inhibited invasion compared to IgG ($P = 0.096$, Fisher's exact test). **(C)** Tumor vasculature. Vessels were examined with CD31 immunohistochemistry (bar = 100 μm). Vascular density was not significantly altered by FG-3019 and/or PEM. N = 6 (IgG), N = 6 (FG-3019), N = 6 (IgG + PEM), N = 6 (FG-3019 + PEM) in (B) and (C); means ± SEM. N is the number of mouse which we used. Pathological specimens were made by each mouse and analyzed. i.p., intraperitoneal injection; i.pl., intrapleural injection; PEM, pemetrexed; N.S., not significant.

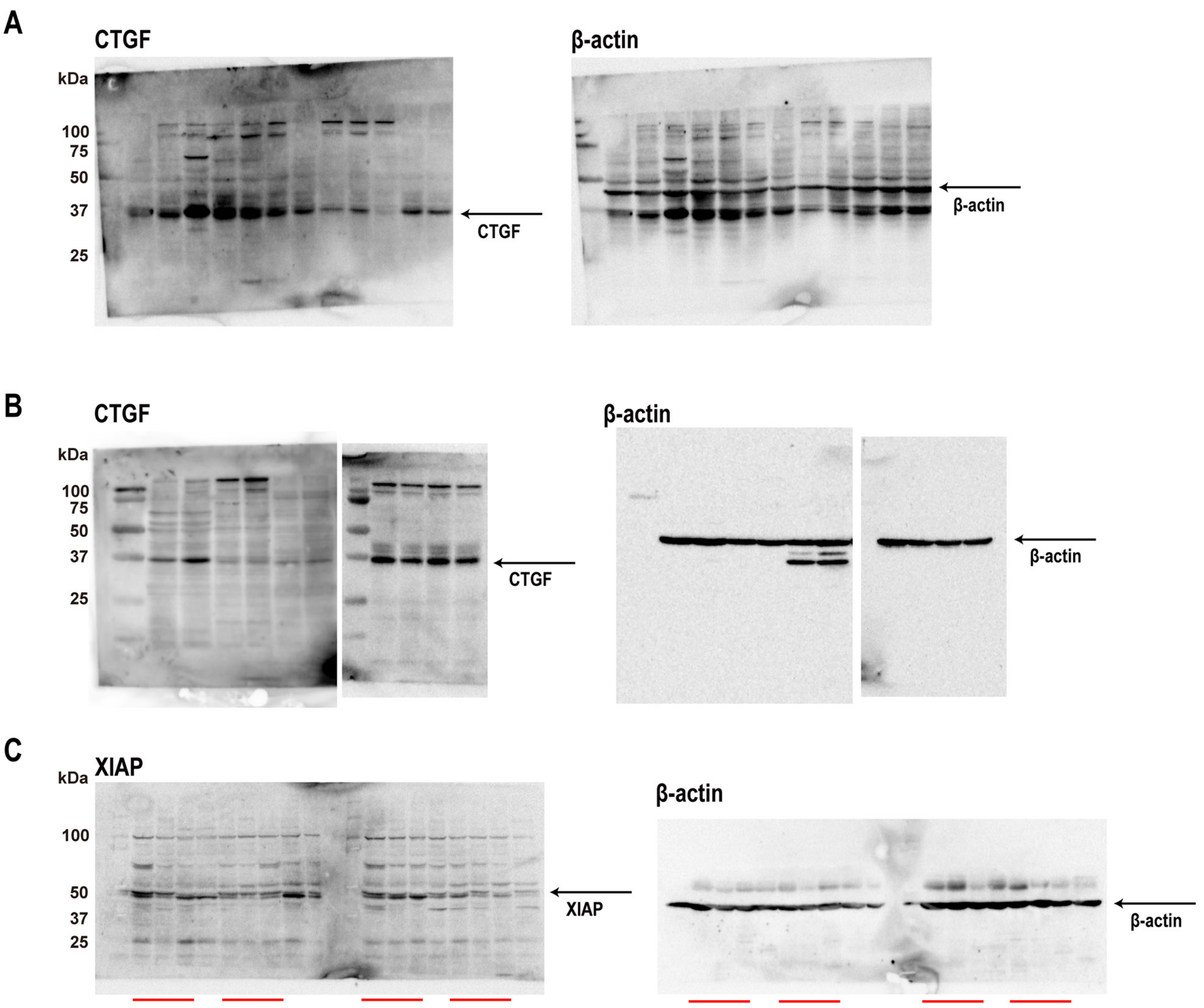

Supplementary Figure 5: Uncropped images of immunoblots. The images from Figure 1A **(A)**, Figure 3D **(B)**, Figure 6A **(C)**. In Figure 6A (C), lanes with red bars are used.
